# Recent Advances in Understanding of the Role of Synuclein Family Members in Health and Disease Volume II

**DOI:** 10.3390/biomedicines14010067

**Published:** 2025-12-29

**Authors:** Natalia N. Ninkina, Vladimir L. Buchman

**Affiliations:** 1 School of Biosciences, Cardiff University, Cardiff CF10 3AX, UK; buchmanvl@cardiff.ac.uk; 2 Research Institute of Pharmacology of Living Systems, Belgorod State National Research University, 308015 Belgorod, Russia

The synuclein family of three short, intrinsically disordered and predominantly neurospecific proteins (α-, β-, and γ-synuclein) [1] has attracted the attention of researchers and clinicians due to their direct or indirect involvement in the molecular pathogenesis of certain neurodegenerative diseases, mainly α-synucleinopathies [2,3,4].

Despite significant progress in our understanding of the role of α-synuclein in the aetiology and pathogenesis of these diseases [5,6], many aspects of this protein disfunction, as well as its normal function in the nervous system and beyond, are still enigmatic [7,8,9,10]. The role of two other members of the synuclein family in these processes is even less clear [11,12,13]. This makes the identification of targets and tools for the efficient combating of pathological processes in the nervous systems of patients with α-synucleinopathies rather difficult.

Therefore, further and more detailed studies on the normal function and malfunction of each member of the synuclein family, employing a variety of different methodological approaches, are timely and important. This Special Issue is devoted to some of such studies.

The interactions of synucleins with other proteins [14,15,16], lipid membranes [17,18,19,20], and various small molecules [21,22] as modifiers of synucleins’ ability and/or propensity to aggregate and, consequently, produce various molecular species toxic to neurons [23,24] has been a focus of many studies because such interactions represent a promising target for the disease-modifying therapy of α-synucleinopaties.

For a number of years, an accumulation of iron was considered a potential trigger of pathological processes, leading to the development and progression of the most common α-synucleinopaty, Parkinson’s disease; therefore, iron chelators could be used in the treatment of this disease [25,26,27]. However, the paper by Huenchuguala and Segura-Aguilar, published in this issue [28], provides recent evidence that this is not the case, and that iron is not an important player in the development of neurodegeneration in Parkinson’s disease.

A growing body of evidence points to the gut–brain axis as one of the main routes of α-synuclein pathology spreading across the body, with the gut endothelial cells being a primary source of pathological species of α-synuclein aggregation [29,30,31,32]. In their paper, Gorecki et al. [33] demonstrated that the presence of α-synuclein in the enteroendocrine cells and their ability to efficiently internalise its preformed filaments (PFFs) made them an early focal point of the pathology spreading and therefore an attractive target for halting this process at the very early stage. Furthermore, the authors show that the poly-Arginine Peptide R18D is able to prevent PPF uptake. making it a promising therapeutic tool.

In contrast, Surguchov et al. [34] discuss the beneficial rather than the harmful properties of amyloidogenic proteins, including synucleins. The main theme of this paper is the potential link between these proteins and infectious diseases, with a particular emphasis on the COVID-19 pandemic.

In a group of papers, Vorobyev and colleagues used electroencephalogram (EEG) studies to address questions related to the role of the synuclein family members in the functional connectivity between brain regions and the dysfunction of this connectivity typical in neurodegenerative conditions. First, they demonstrated that age-related adaptive changes could alleviate problems in EEG coherence, which reflects the functional connectivity between various brain regions, associated with neurodegenerative conditions [35]. Animal models and particularly mouse models were widely used for studying the involvement of synuclens in normal and pathogenic molecular mechanisms, allowing for the uncovering of new, important details [36,37]. Thus, Vorobyev and colleagues assessed EEG coherence in two knockout mouse lines designed to apply different strategies for α-synuclein depletion. The authors found that suppression of EEG coherence was similar in mice with complete germline knockout of the *Snca* gene and those following the inactivation of this gene in adult mice by tamoxifen-induced Cre/LoxP recombination [38]. This research group also studied the suppression of EEG coherence in mice with various combinations of germline knockouts of genes encoding members of the synuclein family, including triple knockout mice lacking all three family members [39]. Their results revealed that depending on what synuclein or combination of synucleins is lost, different functional interactions become suppressed in the mouse brain, suggesting specific roles for each synuclein in the synaptic connectivity between various brain regions.
